# Pyelonephritis in slaughter pigs and sows: Morphological characterization and aspects of pathogenesis and aetiology

**DOI:** 10.1186/1751-0147-52-48

**Published:** 2010-08-12

**Authors:** Louise K Isling, Bent Aalbæk, Malene Schrøder, Páll S Leifsson

**Affiliations:** 1 Department of Veterinary Disease Biology, Faculty of Life Sciences (LIFE), University of Copenhagen, Grønnegårdsvej 15 st., DK-1870 Frederiksberg C, Denmark; 2 Fluisense ApS, Gydevang 42, DK-3450 Allerød, Denmark

## Abstract

**Background:**

Pyelonephritis is a serious disease in pig production that needs to be further studied. The purpose of this study was to describe the morphology, investigate the pathogenesis, and evaluate the aetiological role of *Escherichia coli *in pyelonephritis in slaughtered pigs by concurrent bacteriological, gross and histopathological examinations.

**Methods:**

From Danish abattoirs, kidneys and corresponding lymph nodes from 22 slaughtered finishing pigs and 26 slaughtered sows with pyelonephritis were collected and evaluated by bacteriology and pathology. Based on gross lesions, each kidney (lesion) was grouped as acute, chronic, chronic active, or normal and their histological inflammatory stage was determined as normal (0), acute (1), sub-acute (2), chronic active (3), or chronic (4). Immunohistochemical identification of neutrophils, macrophages, T-lymphocytes, B-lymphocytes, plasma cells, *E. coli *and Tamm-Horsfall protein (THP) in renal sections was performed. The number of *E. coli *and the proportion of immunohistochemically visualized leukocytes out of the total number of infiltrating leukocytes were scored semi-quantitatively.

**Results:**

Lesions in finishing pigs and sows were similar. Macroscopically, multiple unevenly distributed foci of inflammation mostly affecting the renal poles were observed. Histologically, tubulointerstitial infiltration with neutrophils and mononuclear cells and tubular destruction was the main findings. The significant highest scores of L1 antigen^+ ^neutrophils were in inflammatory stage 1 while the significant highest scores of CD79αcy^+ ^B-lymphocytes, IgG^+ ^and IgA^+ ^plasma cells were in stage 3 or 4. Neutrophils were the dominant leukocytes in stage 1 while CD3ε^+ ^T-lymphocytes dominated in stage 2, 3 and 4. Interstitially THP was seen in 82% and 98% of kidneys with pyelonephritis from finishing pigs and sows, respectively. *E. coli *was demonstrated in monoculture and/or identified by immunohistochemistry in relation to inflammation in four kidneys from finishing pigs and in 34 kidneys from sows.

**Conclusions:**

*E. coli *played a significant role in the aetiology of pyelonephritis. Neutrophils were involved in the first line of defence. CD3ε^+ ^T-lymphocytes were involved in both the acute and chronic inflammatory response while a humoral immune response was most pronounced in later inflammatory stages. The observed renal lesions correspond with an ascending bacterial infection with presence of intra-renal reflux.

## Background

Pyelonephritis is a serious disease in pig production causing reduced animal welfare and considerable economic losses due to morbidity and mortality [1-3]. In slaughtered finishing pigs and slaughtered sows, pyelonephritis with variable severity of pelvic lesions is an occasional post mortem finding [4-7]. In addition to the veterinary aspects, porcine pyelonephritis is used as a model of pyelonephritis in humans. However, a detailed pathological characterization of pyelonephritis in pigs is yet to be done as only a few morphological characterizations of porcine pyelonephritis cases have been done and as experimental studies have focused on the cause of renal scarring [8,9]. Identification and location of inflammatory cells in pyelonephritis lesions of different age will improve the understanding of the pathogenesis in pigs and will improve the use of the pig as a model for human pyelonephritis.

Pyelonephritis is generally considered to be caused by ascending bacterial infections and can be either obstructive or non-obstructive. Vesicoureteral reflux (VUR) and intra-renal reflux (IRR) probably play a central role in the pathogenesis. However, the exact role of reflux and bacterial infection is a matter of dispute [10-12]. It has been shown that sterile high-pressure reflux can cause renal lesions in pigs [10,13] and isolation of bacteria from cases of pyelonephritis has not always been possible [1,14]. Previously an immunological response triggered by extravasated Tamm-Horsfall Protein (THP) has been suggested as a cause of renal lesions [8,13,15,16]. The distribution and role of THP in cases of spontaneous porcine pyelonephritis is, however, yet to be finally elucidated.

*E. coli *is one of the most commonly isolated bacteria from sows with pyelonephritis [1,14,17,18], whereas the corresponding bacterial flora of slaughtered finishing pigs has not been thoroughly investigated. However, the role of *E. coli *can be discussed as *Actinobaculum suis*, a specific urinary pathogen, is commonly demonstrated in co-infection with *E. coli *[1,14,17,18] and as the isolation of *E. coli *from urinary tract tissues may be the result of contamination. To our knowledge no studies have visualized *E. coli *directly in relation to renal lesions, and very few researchers have made concurrent bacteriological and pathological studies, which would otherwise improve the aetiological diagnose.

The purpose of the present study was to describe the morphology, investigate the pathogenesis, and evaluate the aetiological role of *E. coli *in pyelonephritis in slaughtered finishing pigs and slaughtered sows in Denmark by concurrent bacteriological, gross and histopathological examinations of renal lesions.

## Materials and methods

### Organs

Kidneys and corresponding lymph nodes from 22 finishing pigs and 26 sows with pyelonephritis slaughtered at Danish abattoirs were sampled based on the presence of renal lesions generally characterized by multiple polyhedral, unevenly distributed, greyish-white foci of inflammation surrounded by a hyperaemic/haemorrhagic rim in acute cases and by the presence of fibrosis in chronic cases [4,7]. Both kidneys were sampled in all cases even if the condition was unilateral.

### Bacteriology

In all but one pig, access to the renal pelvis was made with sterile instruments after searing the kidney surface with a hot metal spatula. Through an incision in the renal parenchyma into the pelvis a swab was taken, plated on blood agar plates (Blood agar base (Oxoid, Basingstoke, Hampshire, United Kingdom), supplemented with 5% sterile bovine blood) and incubated aerobically at 37°C. Except for kidneys from finishing pigs without gross lesions and kidneys from the last seven submitted finishing pigs, an additional bacteriological examination was done. In these cases, one kidney tissue specimen, if possible containing lesions, was sampled and the surface was decontaminated by immersion in boiling water. Subsequently, material from the cut surface of an inflammatory focus was plated on blood agar plates and was incubated aerobically at 37°C to evaluate if the bacterial flora in pelvis and renal tissue were similar. All samples obtained from sows were also incubated anaerobically to permit growth of *A. suis*. Due to methodological reasons anaerobic incubation of samples from finishing pig was not performed. Inoculated plates were read after incubation for 24 h and 48 h. In cases with bacteriological growth of a monoculture, the isolates were identified using standard methods for phenotypic characterization [19].

### Gross pathology, histopathology and immunohistochemistry

All kidneys were sectioned from the free margin to the hilus, to expose cortex, medulla, papillae and pelvis. Gross lesions in kidneys and renal lymph nodes were recorded. Based on the age of gross lesions, kidneys (lesions) were grouped as: acute lesions (A), chronic lesions (presence of fibrosis) (C), chronic active lesions (presence of both acute and chronic lesions) (CA), and normal (N) [4,7].

**Figure 1:**
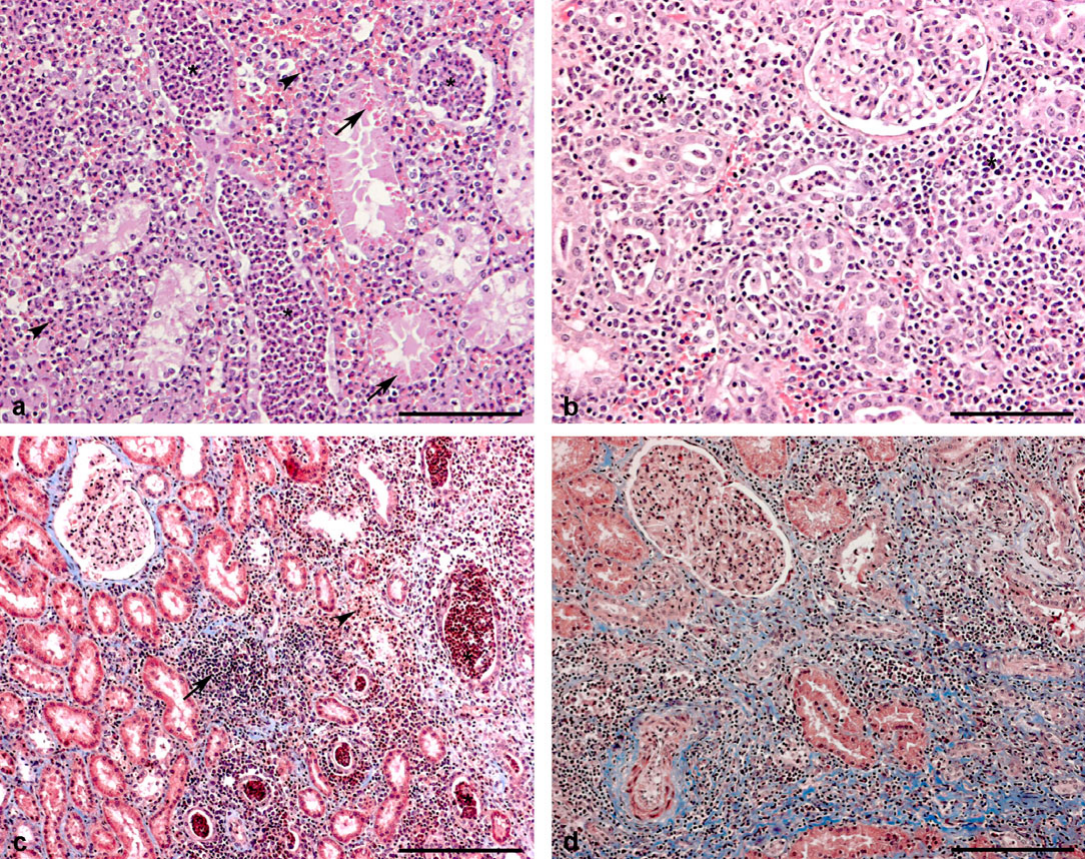
**Histological lesions in porcine kidneys with inflammatory stage 1, 2, 3 and 4**. (a) Section from a kidney with inflammatory stage 1 (acute lesions). Haemorrhage and interstitial cellular infiltration dominated by neutrophils (arrowheads) can be seen. Furthermore tubules dilated with suppurative exudate (asterisks) and tubular destruction are presented. Hyaline droplets are shown in tubular epithelial cells (arrows). Haematoxylin and eosin. Bar = 10 μm. (b) Section from a kidney with inflammatory stage 2 (sub-acute lesions). In contrast to Figure 1a, the interstitial cellular infiltration is dominated by mononuclear cells (asterisks). Haematoxylin and eosin. Bar = 10 μm. (c) Section from a kidney with inflammatory stage 3 (chronic active lesions). Oedema, haemorrhage and interstitial cellular infiltrations consisting of both mononuclear cells (arrow) and neutrophils (arrowhead) can be seen. Furthermore tubules dilated with suppurative exudate (asterisks) are presented. Mild interstitial and periglomerular fibrosis is shown. Masson trichrome. Bar = 20 μm. (d): Section from a kidney with inflammatory stage 4 (chronic lesions). Massive interstitial, periglomerular and perivascular fibrosis can be seen. Furthermore interstitial mononuclear cellular infiltration is shown. Masson trichrome. Bar = 20 μm.

Representative renal samples (centre and poles) and renal lymph nodes were fixed in 10% neutral-buffered formalin for minimum 48 h. Subsequently, samples were routinely processed, embedded in paraffin, sectioned at 2-4 μm, mounted on slides and stained with haematoxylin and eosin [20]. Selected sections were stained with Masson trichrome technique for collagen and with Periodic acid-Schiff for confirmation of connective tissue and goblet cells, respectively [20]. Two to four sections from each kidney were examined systematically and placed into one of the following five groups of inflammatory stages (Figures 1a-d): (0) no pyelonephritic lesions (normal), (1) areas with oedema, hyperaemia, haemorrhage and interstitial cellular infiltrations dominated by neutrophils, in some places forming micro-abscesses, and tubules dilated with suppurative exudates and tubular destruction (acute), (2) as (1) but interstitial cellular infiltrations dominated by mononuclear cells (sub-acute), (3) as (1) and/or (2) with additional presence of mild fibrosis (chronic active), and (4) moderate to massive fibrosis, interstitial mononuclear cellular infiltrations and no or a few interstitial and intratubular neutrophils (chronic). Based on the overall observed histological lesions, each kidney was placed into one of the five inflammatory stages (0-4). Sections from lymph nodes were evaluated for lesions including presence of neutrophils, eosinophils, haemorrhage and hyperaemia.

Immunohistochemical (IHC) detection of CD3ε, CD79αcy, L1 antigen, immunoglobulin (Ig) A, G and M, lysozyme, *E. coli*-antigens and THP was done on at least one representative section from each kidney mounted on adhesive slides (Superfrost^® ^Plus, Menzel-Gläser, Germany) according to table 1. In addition, IHC detection of *E. coli*-antigens was done on sections from lymph nodes of kidneys with a monoculture of *E. coli *and/or IHC positive for *E. coli*-antigens. Sections were heated at 70°C for 15 min and then processed through xylene and rehydrated in graded concentrations of ethanol. Anti-*E. coli *antibody was incubated for 1 h at room temperature (around 20°C), while all other primary antibodies were incubated overnight at 4°C. In renal histological sections, the number of *E. coli *and the proportion of each kind of IHC visualized leukocyte out of the total number of infiltrating leukocytes were scored semi-quantitatively in the following way: none (0), few (<5% of leukocytes) (1), some (>5%-20% of leukocytes) (2), many (>20%-50% of leukocytes) (3) and very many (>50% of leukocytes) (4). The localization and distribution of leukocytes, THP and *E. coli *was investigated.

**Table 1:** Immunohistochemical staining procedures.

Antibody specificity	Clone	**Source/cat. no**.	Dilution^1^	Washing^2^	Blocking of endogenous peroxidase activity^3^	Blocking of unspecific protein binding^4^	Antigen retrieval^5^	Detection^6^	Chromogen^7^/min	Nonsense antibody^8^
Monoclonal										
										
Mouse anti-porcine CD3ε	PPT3	SouthernBiotech, Inc USA/ SB 4510-01	1:1000^a^	TBS +0.5% Triton-X-100	0.6% H_2_O_2_	Ultra V Block^a^	Tris-EGTA^a^	Ultra vision ONE HRP Polymer^a^	DAB^a^/6	X0931
Mouse anti-human CD79αcy	HM57	Dakocytomation, Denmark/ M7051	1:50^c^	TBS +0.5% Triton-X-100	0.6% H_2_O_2_	Ultra V Block^a^	Tris-EDTA^b^	Ultra vision ONE HRP Polymer^a^	DAB^a^/10	X0931
Mouse anti-human monocyte, macrophage, neutrophil	MAC387	Serotec Ltd, UK/MCA874G/ MAC387	1:500^d^	TBS	0.6% H_2_O_2_	Ultra V Block^a^	Tris-EDTA^b^	UltraVision LP large volume detection system HRP polymer^b^	AEC^b^/10	X0931
										
Polyclonal										
										
Goat anti-pig IgG-Fc fragment		Bethyl Laboratories, USA/ A100-104A	1:7000^c^	TBS	0.6% H_2_O_2_	5% rabbit serum^b^	Protease^c^	PAP-goat^c^	DAB^a^/6	I9140
Goat anti-pig IgA		Bethyl Laboratories, USA/ A100-102A	1:4000^c^	TBS	0.6% H_2_O_2_	5% rabbit serum^b^	Protease^c^	PAP-goat^c^	DAB^a^/6	I9140
Goat anti-pig IgM μ- chain specific		Bethyl Laboratories, USA/ A100-100A	1:5000^c^	TBS	0.6% H_2_O_2_	5% rabbit serum^b^	Protease^c^	PAP-goat^c^	DAB^a^/6	I9140
Rabbit anti-human Lysozym		Dakocytomation, Denmark/ A0099	1:200^a^	TBS +0.5% Triton-X-100	3% H_2_O_2_	Ultra V Block^a^	0.1% trypsin^e^	Ultra vision ONE HRP Polymer^a^	DAB^a^/10	X0903
Rabbit anti- *Escherichia Coli*		Dakocytomation, Denmark/ B0357	1:500^e^	TBS	0.6% H_2_O_2_	Ultra V Block^a^	Protease^d^	Ultra vision ONE HRP Polymer^a^	AEC^b^/10	X0903
Sheep anti-human Uromucoid (IgG fraction)		The Binding Site Ltd, UK/ PC071	1:700^b^	TBS +0.5% Triton-X-100	0.6% H_2_O_2_	5% rabbit serum^b^	Protease^c^	AP-sheep/goat^d^	DAB^a^/6	013-000-002

^1 ab ^Diluted in ^a^0.1% or ^b^1% bovine serum albumin (BSA) (A7906, Sigma-Aldrich A/S Denmark) and 0.01% Tween 20 (P-1379, Sigma-Aldrich A/S, Denmark) in Tris-buffered saline 0.05 M Tris, pH 7.6, 0.15 M NaCl (TBS). ^cd^Diluted in ^c^0.1% or ^d^2% BSA in TBS ^e^Diluted in 5% normal swine serum (26250-084, Invitrogen, Denmark) in TBS.

^2^Triton X-100 (PIER28314, VWR - Bie & Berntsen A/S, Denmark).

^3^15 min at room temperatur.

^4 a^Ultra V Block from Ultra vision ONE HRP Polymer (TA-125-UB, Thermo Scientific, USA). ^b^Rabbit serum (X0902, Dakocytomation, Denmark).

^5 ab ^Microwave oven in ^a^Tris-EGTA/^b^Tris-EDTA buffer pH 9 2 × 5 min (watt 700) and 15 min cooling. ^cd ^Protease (P8038, Sigma-Aldrich A/S, Denmark) (^d^0.18-^c^0.36 mg/ml) in TBS followed by incubation in ice cold TBS (pH 7.6) for 2 × min. ^e^Trypsin 1 mg/ml (Sigma-Aldrich A/S, Denmark) in TBS for 2 h at 37°C.

^6 a ^UltraVision ONE large volume detection system HRP polymer (Ready-to-use) (TL-125-HLJ,

Thermo Scientific, USA). ^b^UltraVision LP large volume detection system HRP polymer (Ready-to-use) (TL-125-HL, Thermo Scientific, USA). Both systems were applied according to the manufacturers' instructions. ^c^A three layer horseradish PAP (peroxidase anti-peroxidase) technique using Z0454 (Dakocytomation, Denmark) (1:200 in TBS, 30 min RT) and P1901 (Sigma-Aldrich A/s, Denmark) (1:100 in TBS, 30 min RT). ^d^Donkey Anti-Sheep/Goat immunoglobulins (peroxidase conjugate) (AP360, The Binding Site Ltd, UK).

^7 a^DAB (4150, Kem-En-Tec A/S, Denmark). ^b^AEC-Ready solution from PowerVision+ Poly-AP IHC Kit (Immunovision Techonologies, co). Counterstained with Mayers Haematoxylin 10 sec (LAB00254, VWR - Bie & Berntsen A/S, Denmark).

^8^Togheter with primary antibodies parallel sections were run with nonsense matching species polyclonal (X0903, Dakocytomation, Denmark; I9140, Sigma-Aldrich A/S, Denmark; 013-000-002, Jackson ImmunoResearch, UK) or monoclonal isotype (X0931, X0943, X0944 Dakocytomation, Denmark) antibody in the same protein concentration as the primary antibody.

### Statistics

Fisher's exact test was used for analysis of qualitative data (semi-quantitative scores). Differences were considered statistically significant at *P *< 0.05. All statistical calculations were performed using SAS version 9.1 (SAS Institute, Cary, NC, USA).

## Results

### Gross pathology

**Figure 2:**
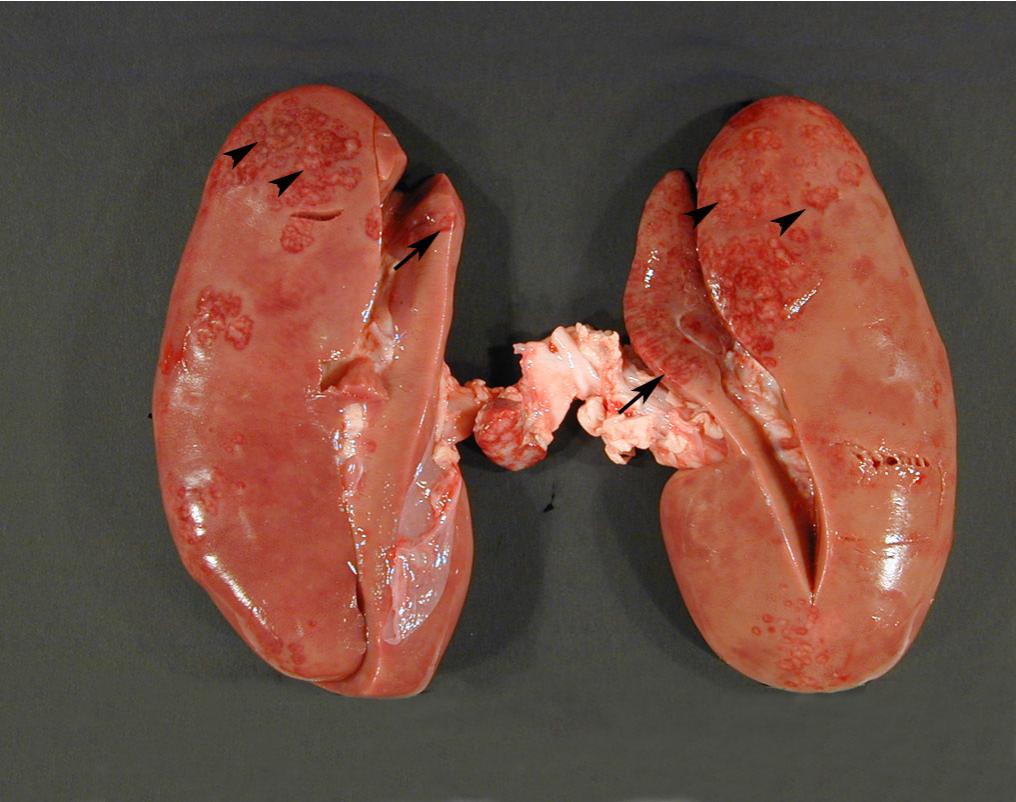
**Gross pathology of acute porcine pyelonephritis**. Kidneys from a slaughtered finishing pig with bilateral acute pyelonephritis. Most of the inflammatory foci are found in the caudal poles (arrowheads). The inflammatory foci extending from the kidney surface through the cortex to medulla as few mm wide streaks and are surrounded by a hyperaemic or haemorrhagic rim (arrows). The renal lymph nodes are enlarged.

Bilateral lesions were observed in 14 finishing pigs (63%) and 19 sows (73%) and acute lesions were shown in 6/8 finishing pigs (75%) and 3/7 sows (43%) with unilateral lesions (table 2). Both kidneys from 10 finishing pigs (45%) and 11 sows (42%) were placed in the same group of gross lesions (table 2). The gross lesions in finishing pigs and sows were similar and consisted of multiple, often confluent, unevenly distributed foci of inflammation usually with a diameter between 3 and 6 mm. Often a more massive affection of the poles was seen (Figure 2). In most cases less than 50% of the kidney parenchyma was affected and usually the lesions encompassed 10 to 20% of an affected kidney. On the kidney surface, acute lesions were seen as round to polyhedral, slightly elevated, greyish-white foci often extending from the surface through the cortex to the medulla as few mm wide streaks and surrounded by a hyperaemic/haemorrhagic rim (Figure 2). Kidneys with many acute lesions were usually enlarged and, when acute lesions were present, varying degrees of oedema and petechiae were found in the underlying pelvic mucosa. In chronic cases, hyperaemia and haemorrhage had subsided and more confluent areas of fibrosis dominated the lesions and renal papillae were often atrophic. On the kidney surface, chronic lesions were slightly depressed below the surrounding surface. Exudates were not found in the pelvis. Bilateral papillary necrosis was seen in three sows. In kidneys with macroscopical lesions, the renal lymph nodes were enlarged (Figure 2) and variable degree of subcapsular blood resorption was a consistent finding in cases with acute lesions.

**Table 2:** Macroscopical group, inflammatory stage and bacteriological results from each kidney pair right/left kidney.

**Kidney pair number** **FP^1^**	**Macro-scopical group^2^**	**Inflam-matory stage^3^**	**Bacteriological result pelvis^4^**	**Bacteriological result kidney parenchyma^4^**	***E. coli *score kidney (IHC)^5^**	**Kidney pair number** **sow**	**Macro-scopical group^2^**	**Inflam-matory stage^3^**	**Bacteriological result pelvis^4^**	**Bacteriological result kidney parenchyma^4^**	***E. coli *score kidney (IHC)^5^**
	
1	A/A	2/2	St/St	St/St	0/0	23	N/A	0/3	*E. coli/E. coli*	St/*E. coli*	0/1
2	CA/CA	3/3	UF/UF	St/St	0/0	24	A/A	2/2	*E. coli/E. coli*	*E. coli/E. coli*	1/2
3	C/N	4/0	St/St	St/ND	0/0	25	A/CA	3/3	*E. coli/*St	St/St	4/0
4	A/N	3/0	UF/UF	St/ND	0/0	26	A/CA	1/3	*E. coli/E. coli*	*E. coli/E. coli*	0/0
5	CA/C	3/3	UF/UF	UF/UF	0/0	27	CA/N	3/0	St/St	St/St	0/0
6	N/A	0/1	UF/*E. coli*	ND/*E. coli*	0/1	28	A/CA	3/1	UF/UF	UF/St	0/0
7	A/A	2/1	St/St	St/St	0/0	29	N/CA	0/3	St/UF	St/St	0/0
8	C/C	4/4	UF/UF	UF/UF	0/0	30	N/A	0/1	St/UF	St/UF	0/1
9	N/A	0/1	UF/UF	ND/*E. coli*	0/2	31	CA/CA	3/3	UF/UF	UF/UF	1/1
10	N/A	0/2	UF/UF	ND/UF	0/3	32	CA/A	1/2	*E. coli/E. coli*	*E. coli/E. coli*	3/4
11	N/A	0/1	St/UF	ND/St	0/0	33	CA/CA	3/3	UF/UF	UF/UF	0/0
12	A/A	2/1	St/UF	St/St	0/0	34	CA/N	3/0	UF/*E. coli*	*E. coli/E. coli*	0/0
13	A/A	3/3	St/St	St/St	0/0	35	CA/CA	3/3	St/UF	St/UF	0/0
14	CA/CA	3/4	UF/UF	UF/UF	1/0	36	CA/CA	3/3	St/St	St/St	0/0
15	A/N	2/0	UF/UF	UF/ND	0/0	37	C/CA	4/3	St/St	*E. coli/E. coli*	0/1
16	A/CA	3/3	ND/ND	ND/ND	0/0	38	CA/CA	3/3	*E. coli/E. coli*	*E. coli/*St	0/0
17	CA/CA	3/3	UF/UF	ND/ND	0/0	39	A/A	3/1	*E. coli/E. coli*	*E. coli/E. coli*	2/4
18	C/CA	3/3	UF/UF	ND/ND	0/0	40	CA/N	3/0	UF/*E. coli*	*E. coli/*St	1/0
19	C/C	3/4	UF/UF	ND/ND	0/0	41	CA/CA	3/3	St/UF	UF/UF	1/0
20	CA/CA	3/3	UF/UF	ND/ND	0/0	42	N/A	0/3	St/UF	St/UF	0/0
21	CA/N	3/0	St/St	ND/ND	0/0	43	A/A	1/2	*E. coli/E. coli*	*E. coli/E. coli*	2/2
22	C/CA	3/3	St/St	ND/ND	0/0	44	CA/CA	3/3	UF/UF	UF/UF	4/1
						45	C/CA	4/3	UF/UF	UF/UF	0/3
						46	C/CA	4/1	UF/*E. coli*	UF/*E. coli*	1/2
						47	A/CA	3/3	*E. coli/E. coli*	*E. coli/E. coli*	2/2
						48	CA/CA	3/4	*E. coli/*UF	*E. coli/*UF	3/1

^1^Finishing pig (FP)

^2^(A) acute lesions, (C) chronic lesions (presence of fibrosis), (CA) chronic active lesions (presence of both acute and chronic lesions), and (N) normal.

^3^(0) normal, (1) acute (2) sub-acute, (3) chronic active, and (4) chronic.

^4^(St) sterile, (UF) unspecific flora, (ND) bacteriological investigation not done, (*E. coli*) *Escherichia coli *isolated as a monoculture.

^5^(0) none, (1) few, (2) some, (3) many, (4) very many.

### Histopathology

The histological lesions in kidneys from finishing pigs and sows were similar. Most kidneys had histological inflammatory stage 3, which was observed in 19 kidneys from 12 finishing pigs and in 30 kidneys from 21 sows (table 2). Inflammatory stage 1 or 2 was observed in 12 kidneys from eight finishing pigs and in 11 kidneys from eight sows and inflammatory stage 4 was observed in five kidneys from four finishing pigs and four kidneys from four sows (table 2). Generally, the cortex was more severely affected than the medulla. Acute lesions were characterized by areas with oedema, hyperaemia, haemorrhage and interstitial cellular infiltrations dominated by neutrophils, in some places forming micro-abscesses. In addition, tubules dilated by a suppurative exudate and tubular destruction were observed (Figure 1a). In older lesions, mononuclear cells dominated the interstitial inflammation (Figures 1b-d). In chronic lesions, variable degree of interstitial fibrosis, as well as fibrosis around vessels and glomeruli, was observed and the suppurative inflammation had subsided (Figure 1d). Perivascular cellular infiltrations primarily with mononuclear cells were commonly seen. Lymphoid follicles were found in 23 kidneys from 19 finishing pigs and in 18 kidneys from 13 sows with pyelonephritis lesions. Hyaline droplets (Figure 1a) were identified in tubular epithelial cells in seven kidneys from five finishing pigs and three kidneys from three sows with pyelonephritis. Papillary necrosis was seen bilaterally in kidneys from three sows. In kidneys with pyelonephritis, variable degree of intraepithelial and subepithelial pelvic cellular infiltrations mainly with mononuclear cells but also neutrophils and eosinophils were observed and pelvic goblet cell metaplasia was commonly seen.

In macroscopically normal kidneys, small numbers of mononuclear cells were identified in six kidneys from finishing pigs and four kidneys from sows and lymphoid follicles were observed in three kidneys from finishing pigs and in one kidney from a sow without gross lesions.

In lymph nodes corresponding to kidneys with pyelonephritis, blood and variable number of neutrophils and eosinophils were often found in the subcapsular and intertrabecular sinuses and there was widespread lymphoid hyperplasia.

### Cellular and Tamm-Horsfall Protein immunohistochemistry

**Figure 3:**
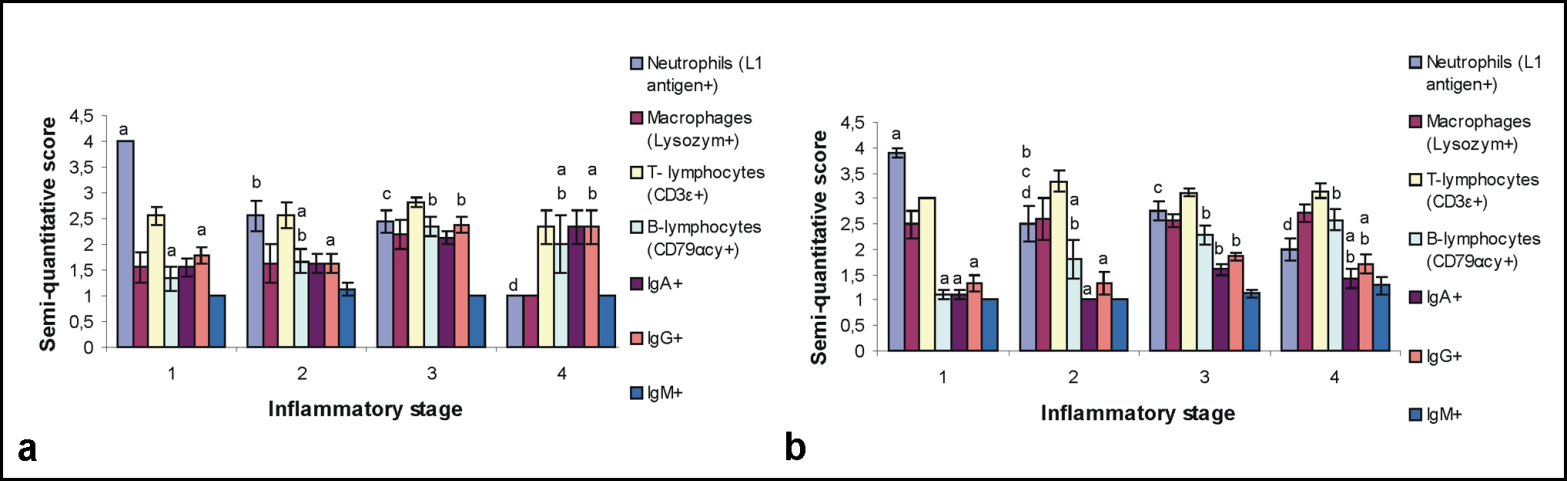
**Score of leukocytes in kidney sections with inflammatory stage 1, 2, 3 and 4**. Mean +/- SEM of semi-quantitative score of leukocytes in kidney sections from slaughtered finishing pigs (a) and slaughtered sows (b) in a given group of inflammatory stage. Different letters (a-d) indicate significant differences in the mean score of a given kind of leukocyte between the four groups.

The results of the semi-quantitatively scored IHC visualized leukocytes in inflammatory stage 1-4 are presented in Figures 3a and 3b. In sections from contra-lateral normal kidneys, only a very few leukocytes, primarily CD3ε^+ ^T-lymphocytes and IgA^+ ^plasma cells, were identified in the interstitium. Higher numbers of all IHC visualized leukocytes were found interstitially in cortex and medulla in kidneys with pyelonephritis compared to contra-lateral normal kidneys. L1 antigen^+ ^neutrophils were the dominant leukocytes in sections of inflammatory stage 1 while CD3ε^+ ^T-lymphocytes were the dominant leukocytes in stages 2, 3 and 4. Most mononuclear cells were CD3ε^+ ^T-lymphocytes. Higher numbers of IgG^+ ^than IgA^+ ^plasma cells were seen and only a very few IgM^+ ^plasma cells were observed.

THP was seen interstitially, mostly in areas with severe inflammation including tubular destruction, in 36 kidneys from finishing pigs (82%) and 51 kidneys from sows with pyelonephritis (98%). Only a very few THP deposits were identified in areas without cellular inflammation and several areas with inflammation without THP deposits were observed.

### Bacteriology and *E. coli *immunohistochemistry

The cultivation results were similar in pelvis and cortex for most kidneys (table 2). *E. coli *was the only bacterium isolated as a monoculture. In finishing pigs, *E. coli *was isolated as a monoculture in two kidneys from two different pigs both of which had unilateral pyelonephritis and belonged to inflammatory stage 1 (table 2). In sows, *E. coli *was isolated as a monoculture in 25 kidneys from 14 sows of which three had unilateral lesions. The kidneys of five of those sows belonged to stage 1, three to stage 2, 13 to stage 3 and one to stage 4 (table 2). From the three sows with unilateral lesions, *E. coli *was also isolated as a monoculture from the contralateral normal kidneys.

**Figure 4:**
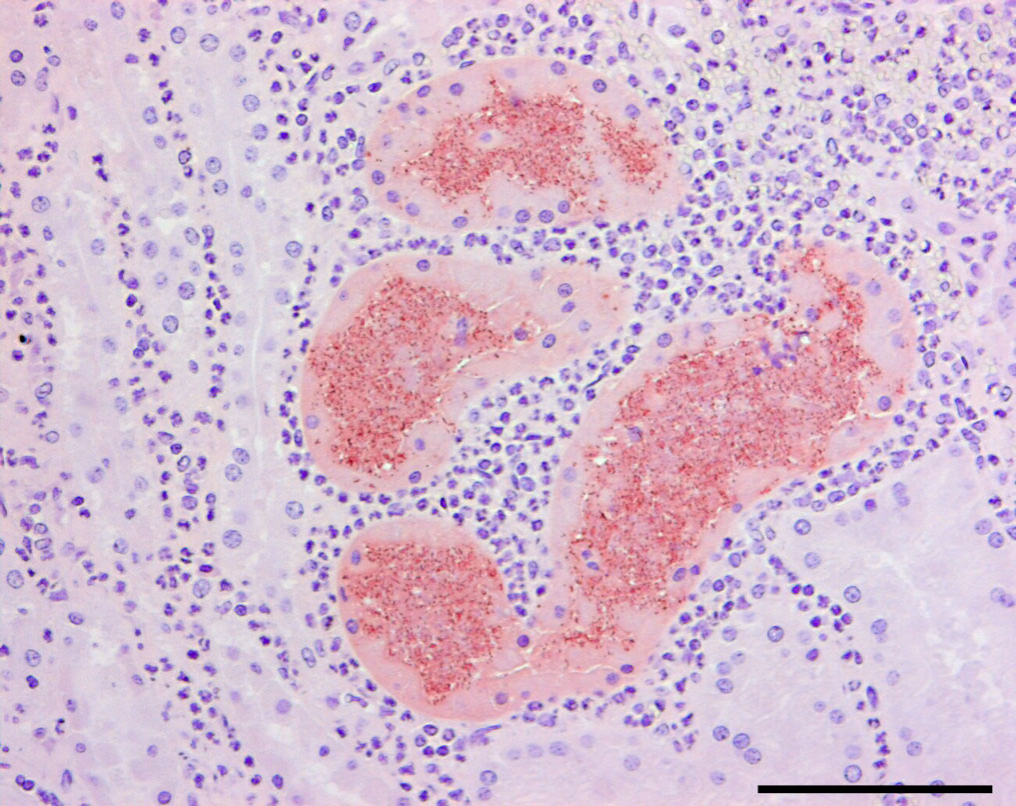
**Immunohistochemical visualisation of *Escherichia coli***. Bacteria with reddish/brown positive immunohistochemical reaction for *E. coli *antibody are seen intratubularly and intracellularly in the tubular epithelium. The affected tubules are surrounded by leukocytes, primarily neutrophils. Bar = 10 μm.

**Figure 5:**
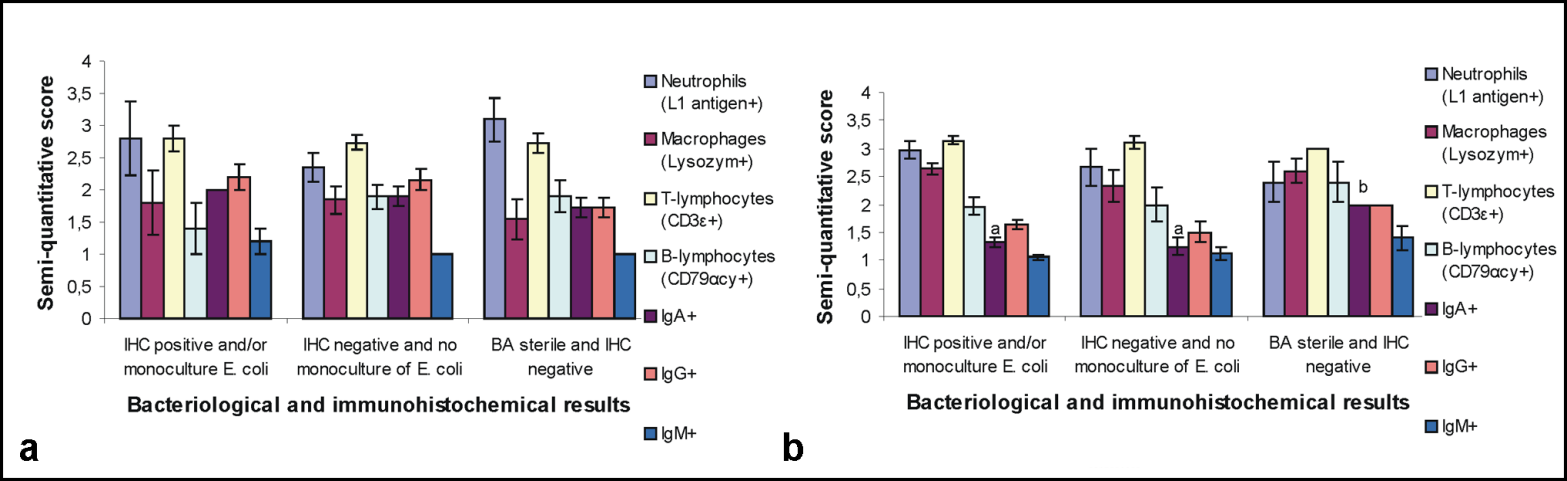
**Score of leukocytes in sections from kidneys with a given bacteriological and immunohistochemical result**. Mean +/- SEM of semi-quantitative score of leukocytes in kidneys from slaughtered finishing pigs (a) and slaughtered sows (b) with a given bacteriological (BA) and immunohistochemical (IHC) result. Different letters (a and b) indicate significant differences in the mean score of a given kind of leukocytes between the three groups.

By IHC staining of *E. coli*-antigens both interstitial and tubular rod-shaped bacteria, either in clusters or solitary were identified (Figure 4). Positive immunoreactions were also recognized intracellularly in macrophages, neutrophils and tubular epithelial cells. A severe inflammatory reaction was nearly always seen in relation to *E. coli*. Four kidneys from four finishing pigs and 25 kidneys from 16 sows showed IHC staining of *E. coli*-antigens (table 2). From those IHC positive kidneys, a monoculture of *E. coli *was isolated from two finishing pig kidneys and from 16 sow kidneys. An unspecific flora, which could include *E. coli*, was observed in all of the IHC positive kidneys where a monoculture of *E. coli *was not isolated. Eleven kidneys from seven finishing pigs and five kidneys from four sows all with pyelonephritis lesions were both IHC negative and bacteriologically sterile (table 2). *E. coli*-antigens were not demonstrated in any corresponding lymph nodes or in any contra-lateral normal kidneys. The association between the presences or absence of *E. coli *demonstrated either by cultivation in monoculture or by IHC and the semi-quantitatively scored leukocytes is presented in Figures 5a and 5b, respectively.

## Discussion

The observed similarity of lesions in finishing pigs and sows supports agreement in aetiology and pathogenesis in the two age groups. Although interpretation of the bacteriological result was complicated by the high frequency of unspecific flora the results suggest an important aetiological role of *E. coli *in the investigated cases as *E. coli *was commonly found in monoculture and/or identified by IHC in relation to renal lesions. In addition, *E. coli *was the only bacterium isolated in monoculture from the kidneys. In kidneys from which a unspecific flora was identified, *E. coli *could probably also have played an aetiological role as an unspecific flora was seen in all IHC *E. coli *positive kidneys from which a monoculture of *E. coli *was not isolated indicating that *E. coli *was most likely part of this unspecific flora. Interestingly, apart from IgA^+ ^plasma cells no significant differences were shown in the mean semi-quantitative score of leukocytes between groups of kidneys with different bacteriological and *E. coli *IHC results, including the sterile IHC negative group, which indicate similarity in pathogenesis no matter the observed bacteriological results and support that a bacteriological infection have been present at one point in all the investigated kidneys. Although *A. suis *is an important pathogen in pyelonephritis in sows [1,17,18], this bacterium was not isolated in this study. This may partly be explained by widely used artificial insemination in Denmark, which reduces the risk of infection [21]. Another reason could be that only slaughtered sows, which were not supposed to have clinical symptoms, were included in the present study. As venereal transmission of *A. suis *is important [22] we do not expect *A. suis *to be a major pathogen in pyelonephritis in finishing pigs. The observation that three contra-lateral normal kidneys were infected with *E. coli *could indicate either contamination or that pyelonephritis were present in some of the renal tissues not investigated histologically. The presence of IHC negative and sterile kidneys with acute lesions corresponds to previous studies describing sterile cases of pyelonephritis in sows [1,14]. Rapid bacterial clearance by host defence, presence of only very low bacterial numbers, insufficient diagnostic methods or lesions caused by unidentified bacterial toxins are possible explanations. An immunological response to THP is, however, not believed to play an important role as interstitially located THP was primarily seen in areas with extensive inflammation suggesting that those extra-tubular deposits were secondary to tubular destruction rather than a primary cause as previously suggested [23]. In addition, the high number of infiltrating neutrophils would not be expected if reflux of sterile urine were solely responsible for the renal lesions [10,13].

Overall the observed gross and histological renal lesions correspond to earlier findings in finishing pigs and sows with pyelonephritis [2,4,7] and to experimental studies of reflux pyelonephritis [24]. IHC identification of leukocytes has, however, not been performed in previous porcine studies. Higher number of all IHC visualized leukocytes was found in kidneys with pyelonephritis compared to kidneys without lesions. As expected the highest mean score of neutrophils was observed in inflammatory stage 1 and the lowest score was in stage 4. The highest mean scores of CD79αcy^+ ^B-lymphocytes, IgG^+ ^and IgA^+ ^plasma cells were observed in stages 3 or 4. L1 antigen^+ ^neutrophils were the dominant leukocytes in kidney sections belonging to inflammatory stage 1 while CD3ε^+ ^T-lymphocytes were the dominant leukocytes in stages 2, 3 and 4. These results show that neutrophils were important in the first line of defence and CD3ε^+ ^T-lymphocytes were suggested to be involved in both the acute and chronic inflammatory response. The importance of T-lymphocytes in Gram-negative infections is not well understood but it is possible that the T-lymphocytes exert a beneficial effect through helper function in the production of protective antibodies or by bactericidal effects [25]. The role of T-lymphocytes in the defence against pyelonephritis has been debated. Studies have shown that mice and rats lacking a functional lymphocyte population did not show significantly reduced resistance to *E. coli *pyelonephritis, thus indicating that T-lymphocytes do not contribute to defence mechanisms and cell damage [26,27]. In contrast, other studies indicate that T-lymphocytes play an important role in the early local response to the infections [28,29]. In the present study, a local humoral immune response with presence of mostly IgG^+ ^but also IgA^+ ^and IgM^+ ^plasma cells was more pronounced in later inflammatory stages. Antibody-mediated immunity has been shown to be crucial in both experimental models and in human cases of pyelonephritis [30]. In a rat pyelonephritis model, IgG, IgA and IgM-producing cells have been observed in renal lesions [28] and abundant numbers of plasma cells have been noted at day 15 of infection [29]. In the present study, neutrophils and lymphocytes were suggested to be involved both in bacterial clearance and in induction of renal injury as tubular destruction was seen in areas with massive cellular infiltration.

The uneven distribution of renal lesions with a predominant affection of the renal poles and the high frequency of unilateral lesions in the present study substantiates the hypothesis of an ascending in contrast to a haematogenous pathogenesis. An ascending pathogenesis is supported by a resemblance to the observed renal lesions in the present study and lesions reported for pigs with experimental ascending reflux pyelonephritis [8,24]. The occurrence of concurrent chronic and acute renal lesions in the majority of the investigated kidneys suggests recurring exposure to the aetiological agent. Presence of a defective vesicoureteral junction causing recurring VUR could explain such inflammation pattern. IRR resulting in introduction of *E. coli *directly into the renal parenchyma with initiation of a tubulointerstitial inflammation can be a way to explain that severe pelvic lesions were not seen in most cases. Investigation of the lower urinary tract to identify cases of cystitis and potentially defects in the vesicoureteral junction would improve the evaluation of both ascending infection and presence of reflux but due to the slaughtering routines collection of bladders was not possible.

## Conclusion

*E. coli *was shown to play a significant role in the aetiology of pyelonephritis in slaughter pigs and sows. Neutrophils were involved in the first line of defence. CD3ε^+ ^T-lymphocytes were found to be involved in both the acute and chronic inflammatory response while a humoral immune response was most pronounced in later inflammatory stages. Neutrophils and lymphocytes were suggested to be involved both in bacterial clearance and in induction of renal injury. The observed renal lesions correspond with ascending bacterial infections with presence of IRR. Extra-tubular THP deposits were probably secondary to renal injury.

## Competing interests

The authors declare that they have no competing interests.

## Authors' contributions

LKI, PSL and MS have made substantial contribution to conception and design of the pathological part of the study and analysis and interpretation of pathological results.

LKI and BA have made substantial contribution to conception and design of the bacteriological part of the study and analysis and interpretation of bacteriological results.

LKI has performed the statistical analysis and drafted the manuscript.

All authors have revised the manuscript critically and approved the final manuscript.
